# Multiphasic CT-based multimodal deep learning model for predicting early hepatocellular carcinoma recurrence following liver transplantation

**DOI:** 10.3389/fonc.2026.1767885

**Published:** 2026-03-27

**Authors:** Zhe Wang, Xi-Ran Li, Zhong-Yi Huang, Jia-He Chu, Kun Liu, Qian Ji

**Affiliations:** 1 Department of Radiology, First Central Hospital of Tianjin Medical University, Tianjin, China; 2 College of Quality and Technical Supervision, Hebei University, Baoding, Hebei, China; 3 College of Medicine, Nankai University, Tianjin, China; 4 Department of Radiology, Tianjin First Central Hospital; Tianjin Institute of Imaging Medicine; Tianjin Key Laboratory of Molecular Diagnosis and Treatment of Liver Cancer, Tianjin, China

**Keywords:** deep learning, hepatocellular carcinoma, liver transplantation, multiphasic CT, recurrence

## Abstract

**Objective:**

This study aimed to develop a multimodal deep learning (MD DL) model integrating multiphasic computed tomography (CT) with clinical and laboratory parameters to predict early recurrence of hepatocellular carcinoma (HCC) following liver transplantation.

**Methods:**

A retrospective analysis was conducted on 147 patients with HCC who underwent liver transplantation at Tianjin First Central Hospital between June 2014 and September 2022. Patients were categorized into recurrence (*n* = 40) and non-recurrence (*n* = 107) groups. Independent risk factors for early recurrence were identified to construct a clinical-imaging model. Deep learning models were developed using both single-phase and multiphasic CT images. High-dimensional imaging features were combined with clinicoradiological parameters to establish the MD DL model. Model performance was evaluated using receiver operating characteristic curves and the DeLong test, while interpretability was assessed through SHapley Additive explanation (SHAP) analysis.

**Results:**

Independent risk factors for early recurrence included platelet count, alpha-fetoprotein levels > 400 ng/mL, ascites, arterial peritumoral enhancement, and portal vein tumor thrombus. The MD DL model achieved area under the curve values of 0.972, 0.885, and 0.985 in the training, validation, and test sets, respectively. These values indicated significantly superior predictive performance compared with other models (all *p* < 0.05). SHAP analysis identified key predictive features contributing to model performance.

**Conclusion:**

The MD DL model integrating multiphasic CT and clinical parameters demonstrated high predictive accuracy for early recurrence of HCC after liver transplantation, with diagnostic performance exceeding that of conventional models.

## 1 Introduction

Hepatocellular carcinoma (HCC) is the most common primary liver malignancy and ranks as the fourth leading cause of cancer-related mortality worldwide (1). Liver transplantation is considered as a curative treatment strategy for patients with early-stage HCC, with a reported 5-year overall survival rate of approximately 75% (2). However, recurrence and metastasis remain the principal factors influencing post-transplant survival (3). The incidence of recurrence has been reported at approximately 10% to 20% (2), with a median survival of only 1 year following recurrence or metastasis (4). Patients with a low risk of recurrence generally achieve more favorable outcomes after liver transplantation; therefore, accurate prediction of postoperative recurrence risk is of clinical importance for guiding treatment decisions.

Recent studies have applied radiomics and deep learning approaches to predict recurrence following HCC resection or transplantation. Zhao et al. constructed a nomogram incorporating computed tomography (CT)-based radiomics features to predict early recurrence after liver transplantation, although only arterial-phase tumor information was included. Mu et al. constructed a deep learning model based on multiparametric magnetic resonance imaging (MRI) to predict early HCC recurrence after liver transplantation (5–9). Meng et al. reported that a multiphase fusion MRI-based deep learning model provided superior diagnostic performance compared with single-phase models (arterial, portal venous, or hepatobiliary phases) in patients undergoing hepatectomy (10). Although multiphasic contrast-enhanced CT is widely used in comprehensive preoperative assessment for liver transplantation, no deep learning model using multiphasic CT has yet been developed to predict early HCC recurrence after transplantation.

The objective of this study was to develop a multimodal deep learning (MD DL) model integrating multiphasic CT imaging with clinical and conventional imaging features for noninvasive preoperative prediction of early HCC recurrence after liver transplantation. This approach is intended to optimize recipient selection, reduce unnecessary donor liver utilization, and improve patient survival and quality of life.

## 2 Materials and methods

### 2.1 Study participants

This retrospective study included patients with HCC who underwent liver transplantation at Tianjin First Central Hospital between June 2014 and September 2022.

The inclusion criteria were: (i) age ≥ 18 years; (ii) availability of complete clinical and laboratory data; (iii) first-time liver transplantation with multiphasic abdominal CT performed within 1 month before surgery; (iv) postoperative pathological confirmation of HCC; and (v) a minimum follow-up period of 2 years.

**Figure 1:**
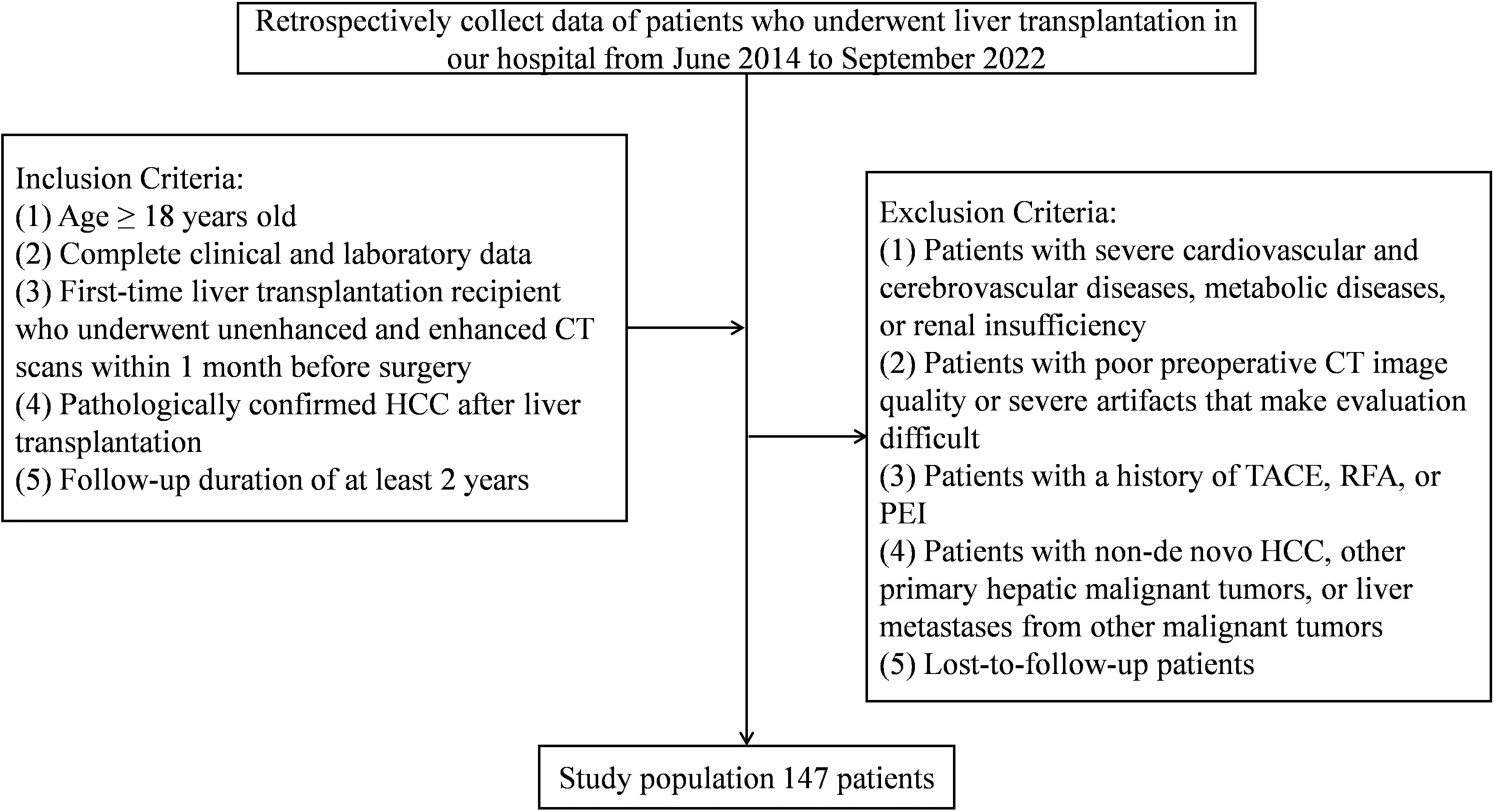
Flowchart of patient inclusion and exclusion criteria.

The exclusion criteria were: (i) severe cardiovascular disease, cerebrovascular disease, metabolic disorders, or renal insufficiency; (ii) poor-quality preoperative CT images with substantial artifacts; (iii) previous treatment with transarterial chemoembolization (TACE), radiofrequency ablation (RFA), or percutaneous ethanol injection (PEI); (iv) recurrent HCC, other primary hepatic malignancies, or hepatic metastases; and (v) loss to follow-up. In total, 147 patients met the criteria and were included. The patient selection process is presented in **Figure 1**.

### 2.2 Collection of clinical and laboratory parameters

Baseline demographic and clinical characteristics were collected, including sex, age, date of CT examination (the most recent preoperative scan, typically within 1 month before surgery), date of liver transplantation, and pathological findings. Laboratory parameters obtained from the most recent preoperative assessment included albumin, total bilirubin, creatinine, platelet count (PLT), prothrombin time, international normalized ratio, neutrophil count (NEUT), lymphocyte count, neutrophil-to-lymphocyte ratio, hepatitis B surface antigen or hepatitis C virus antibody, alpha-fetoprotein (AFP), alanine aminotransferase (ALT), aspartate aminotransferase (AST), and gamma-glutamyl transferase (GGT).

Early HCC recurrence was defined as recurrence within 12 months after liver transplantation.

### 2.3 CT acquisition protocol

All examinations were conducted using multi slice CT, including non-enhanced and four-phase contrast-enhanced liver imaging. Contrast medium was administered via the antecubital vein. For arterial-phase acquisition, a threshold-triggering technique was applied at 150 Hounsfield units (HU), with monitoring at the abdominal aorta near the hepatic hilum. Scanning commenced 20 seconds after the attenuation of the abdominal aorta reached 150 HU, using a bolus-tracking method. Portal venous-phase images were acquired 20 seconds after the arterial phase, venous-phase images 15 seconds after the portal venous phase and delayed-phase images 200 seconds after the venous phase. Detailed scanning parameters are summarized in **Table 1**.

**Table 1:** CT scanner parameters.

Parameters	Revolution CT	Aquilion ONE CT	SOMATOM definition flash CT
Tube voltage (kV)	120	120	120
Tube current	auto	auto	auto
Detector collimation (mm)	80×0.625	80×0.5	128×0.6
	350-400	350-400	350-400
Scan slice thickness (mm)	5	5	5
Slice interval (mm)	5	5	5
Reconstruction slice thickness (mm)	1.25	1	1.2
Matrix	256×256	256×256	256×256
Gantry rotation speed (r/s)	0.5	0.5	0.5
Pitch	0.992	0.9	0.8

### 2.4 Assessment of conventional imaging features

**Figure 2:**
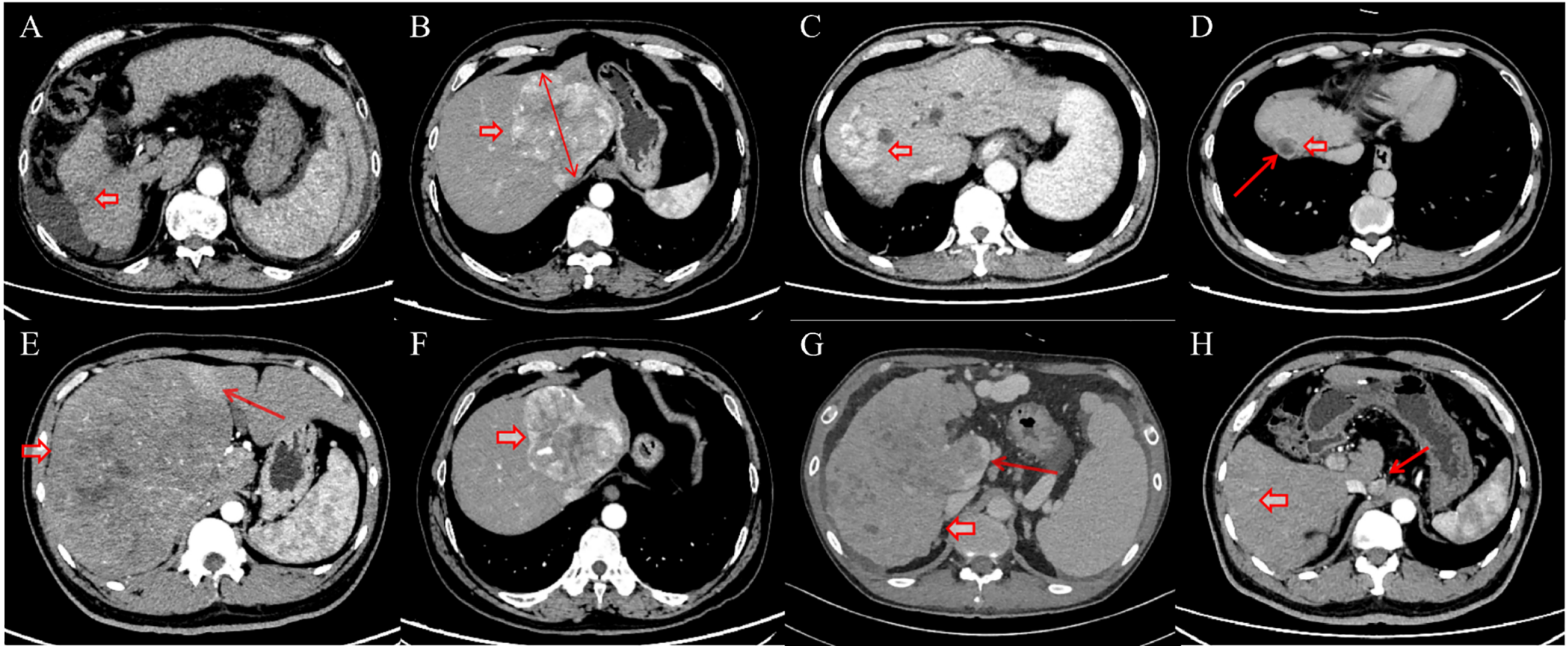
Representative CT images depicting assessment of conventional imaging features in hepatocellular carcinoma (HCC). Red short arrows: HCC. **(A)** Ascites and liver cirrhosis; **(B)** Measurement of maximum tumor diameter (without capsule); **(C)** Irregular tumor margin; **(D)** Tumor capsule enhancement in the delayed phase with smooth margin; **(E)** Arterial peritumoral enhancement; **(F)** Intratumoral arteries and intratumoral necrosis; **(G)** Portal vein tumor thrombus in the main trunk and right branch; **(H)** Enlarged retroperitoneal lymph nodes with heterogeneous enhancement.

Two radiologists with 5 and 7 years of experience in abdominal imaging independently reviewed the preoperative CT images. Both radiologists were blinded to clinical data, laboratory data, and recurrence status. Discrepancies were resolved by a senior radiologist with more than 10 years of abdominal imaging experience. The evaluated imaging features included ascites, liver cirrhosis, maximum tumor diameter, tumor margin, capsule, arterial peritumoral enhancement, intratumoral arteries, intratumoral necrosis, portal vein tumor thrombus, abdominal lymph node metastasis (**Figure 2**).

### 2.5 Construction of the clinical-imaging model

Logistic regression analysis was used to identify independent risk factors for early recurrence of HCC after liver transplantation. A clinical-imaging model was constructed using these independent predictors.

### 2.6 Construction of the deep learning model

#### 2.6.1 Delineation of HCC lesions

Two radiologists with 5 and 7 years of experience in abdominal imaging independently delineated regions of interest (ROIs) on non-enhanced, arterial-phase, portal venous-phase, venous-phase, and delayed-phase CT images at the slice containing the largest lesion. Delineation followed the 2020 expert consensus on CT and MRI annotation of focal liver lesions (11). The radiologists were blinded to clinical data, laboratory findings, and recurrence status. In patients with multiple lesions, the largest lesion was delineated. For lesions greater than 5 cm in diameter, additional slices with distinct enhancement patterns centered on the maximal cross-section were delineated to maintain consistency of ROI size and location across all phases. In total, 406 lesion slices were obtained from 147 patients, with each slice containing 5 ROIs corresponding to the 5 CT phases.

#### 2.6.2 Baseline model development

**Figure 3:**
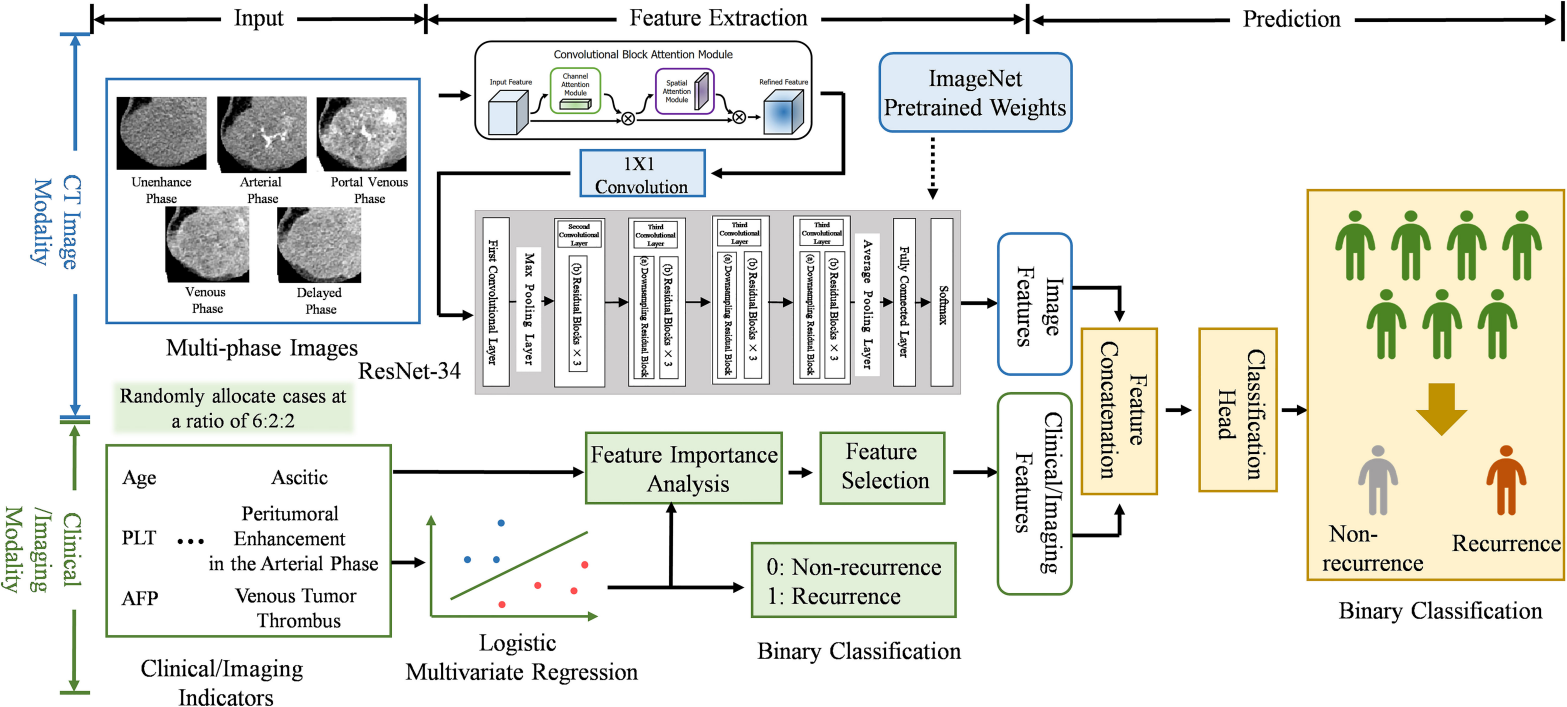
Workflow of multimodal deep learning model construction.

Residual network (ResNet-34) was adopted as the baseline network architecture based on its strong feature extraction capabilities and reduced risk of overfitting in small datasets. A convolutional block attention module was incorporated into the ResNet-34 architecture to fuse multiphase image data into a unified feature representation. To meet the input requirements of ResNet-34, 1 × 1 convolution operations were applied to efficiently map the stacked five-channel grayscale images (representing the five CT phases) into three channels. ImageNet-pretrained weights were used to initialize ResNet-34 during the development of both unimodal and MD DL models. The model development process is presented in **Figure 3**.

#### 2.6.3 Development of unimodal deep learning models

Considering the diagnostic importance of arterial-phase and portal venous-phase images in HCC, unimodal deep learning models were constructed using arterial-phase images, portal venous-phase images, and multiphase images (incorporating all five phases: non-enhanced, arterial, portal venous, venous, and delayed). These models were designated as the arterial-phase deep learning (AP DL) model, portal-phase deep learning (PP DL) model, and multiphase deep learning (MP DL) model, respectively.

Detailed settings for deep learning model training are as follows. The unimodal ResNet-34 model was trained with the Adam optimizer, a batch size of 32, and 30 epochs. A cosine annealing learning rate scheduler was employed with an initial learning rate of 10–^3^ and a minimum learning rate of 10^-6^. L2 regularization (weight decay = 10^-3^) and dropout layers in the feature extractor and fully connected layers were adopted to mitigate overfitting. For the multimodal ResNet-34 model, the optimizer, batch size, regularization, and dropout settings were unchanged, while the training epoch was set to 10 and the initial learning rate to 10^-4^. All hyperparameters were optimized via grid search according to the best performance on the validation set, and the best model was saved for final evaluation on the independent test set. The cross-entropy loss function was used for model training. During training, data augmentation including random rotation, flipping, horizontal translation, and scaling was applied to the training images. To ensure consistency, identical transformation parameters were used across all five phases for each subject. Overfitting was monitored by tracking the validation loss and controlled by dropout and L2 regularization.

#### 2.6.4 Development of the MD DL model

High-dimensional features extracted from imaging modalities were combined with clinical and conventional imaging parameters from tabular data. The combined feature vector was subsequently input into a fully connected classification layer to generate the final classification output. This integrated framework was designated as the MD DL model.

#### 2.6.5 Interpretability analysis of the MD DL model using SHAP

SHAP (SHapley Additive exPlanation) analysis was used to approximate Shapley values through feature sampling and quantify the contribution of each feature to the model output. As image encoders such as ResNet typically generate thousands of high-dimensional features, both absolute and mean values were used to compute feature importance in this analysis.

### 2.7 Statistical analysis

All statistical analyses were conducted using SPSS version 26.0 (IBM, Armonk, NY) and R version 4.4.2 (R Foundation for Statistical Computing, Vienna, Austria). The Kolmogorov-Smirnov test was applied to assess the normality of continuous variables. Normally distributed variables are expressed as mean ± standard deviation ( $\bar{\text{x}}$ ± s), whereas non-normally distributed variables are expressed as median with interquartile range M (P25, P75). Differences between recurrence and non-recurrence groups in clinical and imaging features were evaluated using independent-sample *t*-tests, chi-squared tests or Fisher’s exact tests, and Mann-Whitney U tests, as appropriate.

Logistic regression analysis was used to examine associations between clinical and imaging features and early HCC recurrence after liver transplantation, forming the basis for the clinical-imaging model. Receiver operating characteristic curve analysis and the DeLong test were used to evaluate and compare the predictive performance of the models. A two-sided *p* value < 0.05 was considered statistically significant.

## 3 Results

### 3.1 Comparison of clinical, laboratory, and conventional imaging characteristics between recurrence and non-recurrence groups

A total of 147 patients were included, with 107 in the non-recurrence group (72.8%) and 40 in the recurrence group (27.2%). All patients were randomly assigned into training (*n* = 88), validation (*n* = 29), and test (*n* = 30) groups using stratified random sampling at a 6:2:2 ratio.

Significant differences between recurrence and non-recurrence groups were observed in sex, PLT, NEUT, AFP, ALT, AST, and GGT (*p* <.05). Maximum tumor diameter was also significantly greater in the recurrence group compared to the non-recurrence group (*p* < 0.05). Significant differences were further observed in ascites, liver cirrhosis, arterial peritumoral enhancement, intratumoral arteries, intratumoral necrosis, and portal vein tumor thrombus (*p* < 0.05). No significant differences were observed for the remaining variables (*p* > 0.05) (**Table 2**).

**Table 2:** Comparison of clinical, laboratory, and conventional imaging features between recurrence and non-recurrence groups.

Clinical/imaging features	Non-recurrence group	Recurrence group	*t/χ2/Z*	*P*
Sex (Female/Male)	15/92 (14.0%,86.0%)	1/39 (2.5%,97.5%)	0.003	0.046
Age (years)	56.06 ± 8.14	55.45 ± 8.47	0.397	0.692
ALB (g/L)	34.50 (29.80,42.70)	36.60 (32.03,39.45)	-0.914	0.361
TBIL (μmol/L)	28.20 (17.35,51.98)	29.96 (20.91,78.44)	-0.590	0.555
CREA (μmol/L)	67.00 (58.00,75.40)	69.50 (58.63,80.50)	-0.525	0.600
PLT (10^9^/L)	64.00 (40.00,106.00)	116.00 (77.25,179.00)	-4.148	<0.001
PT (s)	16.40 (13.70,20.50)	16.30 (14.10,22.38)	-0.372	0.710
INR	1.35 (1.17,1.68)	1.30 (1.16,1.55)	-0.457	0.648
NEUT (10^9^/L)	2.35 (1.49,4.05)	3.76 (2.18,5.23)	-2.331	0.020
LY (10^9^/L)	0.75 (0.47,1.06)	0.84 (0.44,1.22)	-0.696	0.486
NLR	3.04 (1.87,6.32)	3.68 (2.42,7.21)	-1.214	0.225
HBsAg/HCVAb	26/81 (24.3%,75.7%)	9/31 (35.0%,77.5%)	0.052	0.820
AFP (≤400 ng/mL/>400 ng/mL)	92/15 (86.0%,14.0%)	92/15 (86.0%,14.0%)	27.718	<0.001
ALT (IU/L)	34.30 (20.90,68.10)	55.05 (37.23,116.70)	-2.807	0.005
AST (IU/L)	67.95 (25.70,82.70)	67.95 (36.45,190.23)	-2.376	0.017
GGT (IU/L)	47.00 (23.80,89.00)	101.00 (40.50,149.50)	-3.389	0.001
Ascites (Absent/Present)	63/44 (58.9%,41.1%)	11/29 (27.5%,72.5%)	11.468	0.001
Liver cirrhosis (Absent/Present)	7/100 (6.5%,93.5%)	7/33 (17.5%,82.5%)	4.057	0.044
Maximum tumor diameter (cm)	2.70 (1.90,4.30)	6.20 (3.03,11.18)	-4.900	<0.001
Tumor margin (Clear/Subtle/Unclear)	34/18/55 (31.8%,16.8%,51,4%)	19/7/14 (47.5%,17.5%,35.0%)	3.673	0.159
Capsule (Absent/Present)	77/30 (72.0%,28.0%)	32/8 (80.0%,20.0%)	0.981	0.322
Arterial peritumoral enhancement (Absent/Present)	104/3 (97.2%,2.8%)	24/16 (60.0%,40.0%)	35.793	<0.001
Intratumoral arteries (Absent/Present)	74/33 (69.2%,30.8%)	11/29 (27.5%,72.5%)	20.719	<0.001
Intratumoral necrosis (Absent/Present)	50/57 (46.7%,53.3%)	10/30 (25.0%,75.0%)	5.691	0.017
Portal vein tumor thrombus (Absent/Present)	98/9 (91.6%,8.4%)	21/19 (52.5%,47.5%)	28.851	<0.001
Abdominal lymph nodes (Absent/Present)	100/7 (93.5%,6.5%)	34/6 (85.0%,15.0%)	2.584	0.108

ALB, albumin; TBIL, total bilirubin; CREA, creatinine; PLT, platelet; PT, prothrombin time; INR, international normalized ratio; NEUT, neutrophil count; LY, lymphocyte count; NLR, neutrophil-to-lymphocyte ratio; HBsAg, hepatitis B surface antigen; HCVAb, hepatitis C virus antibody; AFP, alpha-fetoprotein; ALT, alanine aminotransferase; AST, aspartate aminotransferase level; GGT, gamma-glutamyl transferase. p < 0.05 indicates statistical significance.

### 3.2 Development and evaluation of predictive models for early HCC recurrence after liver transplantation

#### 3.2.1 Clinical-imaging model

Logistic regression analysis identified elevated PLT, AFP > 400 ng/mL, ascites, larger tumor diameter, arterial peritumoral enhancement, intratumoral arteries, intratumoral necrosis, and portal vein tumor thrombus as significant predictors of early HCC recurrence after liver transplantation (*p* < 0.05). Among these, PLT, AFP > 400 ng/mL, ascites, arterial peritumoral enhancement, and portal vein tumor thrombus were independent risk factors (*p* < 0.05) (**Table 3**). These independent variables were incorporated into the clinical-imaging model and subsequently integrated into the MD DL framework.

**Table 3:** Univariate and multivariate logistic regression analysis of risk factors for recurrence.

Parameters	Univariate analysis			Multivariate analysis		
	OR	95%CI	*P* ^1^	OR	95%CI	*P* ^2^
Sex (Female/Male)	6.539	0.812-49.820	0.078			
Age (years)	0.991	0.948-1.036	0.690			
ALB(g/L)	0.998	0.984-1.012	0.764			
TBIL(μmol/L)	1.003	1.000-1.005	0.061			
CERA(μmol/L)	0.999	0.994-1.004	0.628			
PLT (10^9^/L)	0.929	0.499-1.729	0.816			
PT(s)	1.012	1.008-1.017	<0.001	1.011	1.001-1.022	0.038
INR	1.070	0.961-1.191	0.217			
NEUT (10^9^/L)	1.483	0.770-2.855	0.293			
LY (10^9^/L)	0.998	0.971-1.025	0.874			
NLR	1.016	0.966-1.096	0.531			
HBsAg/HCVAb	1.106	0.466-2.622	0.820			
AFP>400ng/mL	8.298	3.614-19.052	<0.001	3.497	1.040-11.760	0.043
ALT(IU/L)	1.000	0.999-1.001	0.974			
AST(IU/L)	1.000	0.999-1.001	0.923			
GGT(IU/L)	1.002	1.000-1.0005	0.101			
Ascites	3.775	1.707-8.348	0.001	3.144	1.086-9.108	0.035
Liver cirrhosis	0.330	0.108-1.010	0.052			
Maximum tumor diameter (cm)	1.375	1.202-1.572	<0.001			
Tumor margin	0.675	0.450-1.013	0.058			
Capsule	0.642	0.266-1.550	0.324			
Arterial peritumoral enhancement	23.111	6.232-85.702	<0.001	6.649	1.178-37.542	0.032
Intratumoral arteries	5.912	2.640-13.239	<0.001			
Intratumoral necrosis	2.632	1.170-5.917	0.019			
Portal vein tumor thrombus	8.776	3.570-21.572	<0.001	4.083	1.128-14.787	0.032
Abdominal lymph nodes	2.521	0.792-8.024	0.118			

ALB, albumin; TBIL, total bilirubin; CREA, creatinine; PLT, platelet; PT, prothrombin time; INR, international normalized ratio; NEUT, neutrophil count; LY, lymphocyte count; NLR, neutrophil-to-lymphocyte ratio; HBsAg, hepatitis B surface antigen; HCVAb, hepatitis C virus antibody; AFP, alpha-fetoprotein; ALT, alanine aminotransferase; AST, aspartate aminotransferase level; GGT, gamma-glutamyl transferase; OR, odds ratio; CI, confidence interval. p1 and p2 represent p values from univariate and multivariate logistic analyses, respectively. p < 0.05 indicates statistical significance.

#### 3.2.2 Predictive performance and comparison of clinical-imaging, unimodal, and MD DL models

In the training and validation groups, the area under the curve (AUC, 95% confidence interval [CI]) of the clinical-imaging model was 0.886 (0.808–0.950) and 0.696 (0.400–0.942), respectively. Both values were lower than those of other models. In the test group, the AUC of the clinical-imaging model was 0.825 (0.633–0.971).

**Figure 4:**
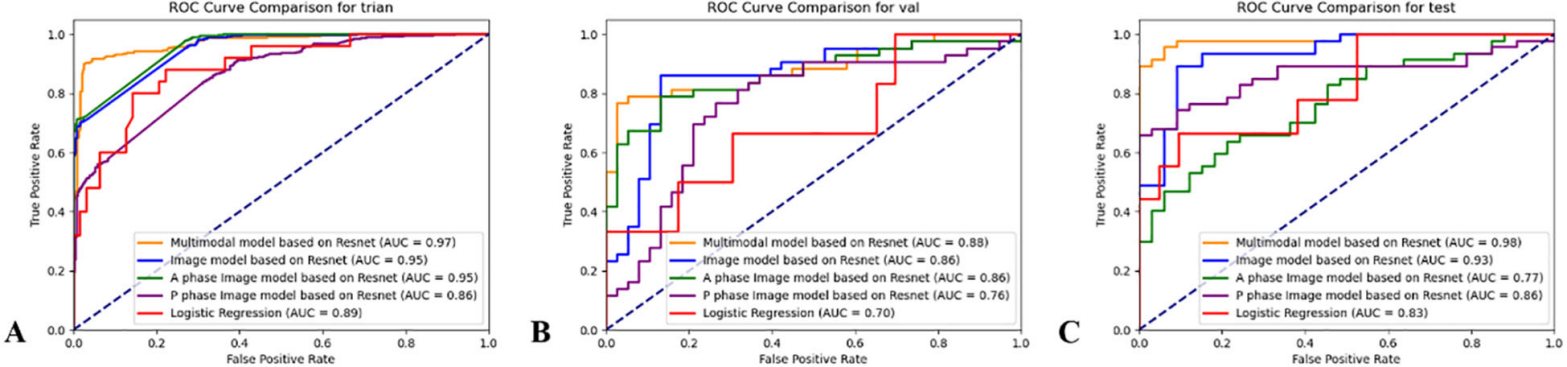
ROC curves of the clinical-imaging model, AP DL model, PP DL model, MP DL model, and MD DL model in training, validation, and test groups. **(A)** Training group; **(B)** Validation group; **(C)** Test group.

Unimodal deep learning models developed from CT images achieved high AUCs in the training group. The AUCs (95% CI) of the AP DL, PP DL, and MP DL models were 0.952 (0.304–0.954), 0.863 (0.730-0.864), and 0.945 (0.935–0.960), respectively. However, the predictive performance of the AP DL and PP DL models reduced in the validation and test groups, indicating limited generalizability. The MP DL model maintained stable performance, with AUCs of 0.857 (0.745–0.968) in the validation group and 0.934 (0.872–0.981) in the test group. The MD DL model demonstrated superior predictive performance across all groups. Its AUCs (95% CI) were 0.972 (0.961–0.981) in the training group, 0.885 (0.809–0.956) in the validation group, and 0.985 (0.959–1.000) in the test group. The MD DL model also achieved the highest sensitivity and specificity of 0.915 and 0.977, respectively, in the test group. DeLong test results indicated that the diagnostic performance of the MD DL model was significantly higher compared with other models in the test group (*p* = 0.017, < 0.001, 0.003, and 0.030, respectively) (**Figure 4**; **Table 4**).

**Table 4:** Predictive performance of clinical-imaging, unimodal, and multimodal deep learning models.

Dataset	Model	Sensitivity	Specificity	Accuracy	F1	AUC	95%CI
Training Set	Clinical-imaging	0.698	0.936	0.750	0.510	0.886	0.808-0.950
	AP DL	0.720	0.964	0.844	0.824	0.952	0.304-0.954
	PP DL	0.696	0.878	0.744	0.615	0.952	0.730-0.864
	MP DL	0.701	0.956	0.832	0.810	0.945	0.935-0.960
	MD DL	0.904	0.960	0.932	0.931	0.972	0.961-0.981
Validation Set	Clinical-imaging	0.478	0.916	0.552	0.364	0.696	0.400-0.942
	AP DL	0.907	0.661	0.704	0.765	0.865	0.780-0.941
	PP DL	0.767	0.767	0.753	0.767	0.767	0.650- 0.870
	MP DL	0.860	0.881	0.864	0.884	0.857	0.845- 0.968
	MD DL	0.814	0.778	0.778	0.795	0.885	0.809-0.956
Test Set	Clinical-imaging	0.619	0.813	0.633	0.545	0.825	0.633-0.971
	AP DL	0.872	0.695	0.700	0.774	0.770	0.666-0.972
	PP DL	0.681	0.970	0.800	0.80	0.864	0.767-0.939
	MP DL	0.863	0.929	0.830	0.876	0.934	0.872-0.981
	MD DL	0.915	0.977	0.938	0.945	0.945	0.959-1.000

AP DL, arterial phase unimodal deep learning; PP DL, portal venous phase unimodal deep learning; MP DL, multiphasic unimodal deep learning; MD DL, multimodal deep learning; AUC, area under the curve; CI, confidence interval. *p* < 0.05 indicates statistical significance.

#### 3.2.3 Interpretability analysis of the MD DL model using SHAP

As presented in **Table 5**, the MD DL model produced a greater number of features with an average contribution equal to zero per sample, indicating that the model concentrated more effectively on features relevant to recurrence prediction while reducing the impact of redundant features. Additionally, in the MD DL model, clinical and conventional imaging characteristics contributed more substantially than high-dimensional features derived from multiphasic CT data. This finding indicates that in assessing recurrence risk, non-imaging information, including clinical and conventional imaging parameters, provides essential and complementary value beyond high-dimensional CT features.

**Table 5:** Feature contribution in unimodal and multimodal deep learning models.

Model	Average statistical contribution of unimodal features per sample	<0 Count	=0 Count	>0 Count	Average absolute contribution
MP DL	High-dimensional imaging features	249.22	56.34	206.44	0.005804
MD DL	Non-imaging features	147.25	229.84	134.91	0.003120
	High-dimensional imaging features	58.99	397.98	55.04	0.000412

## 4 Discussion

HCC is a highly complex and heterogeneous malignancy characterized by distinct microscopic, macroscopic, and molecular features. Multiphasic CT, which is widely used for preoperative assessment in liver transplantation, facilitates accurate detection of critical tumor characteristics. ResNet architectures can effectively capture complex imaging patterns from CT scans while reducing the vanishing gradient problem.

In this study, clinical data, multiphasic CT, and deep learning were integrated to construct a MD DL model based on ResNet architecture. The aim was to achieve effective, noninvasive prediction of early HCC recurrence after liver transplantation and to provide precise auxiliary information for clinical decision-making.

Prior studies indicated that tumor biomarkers, tumor size, heterogeneity, differentiation, and microvascular invasion (MVI) are important factors influencing recurrence after liver transplantation (8, 10, 12). The current findings identified PLT, AFP, ascites, arterial peritumoral enhancement, and portal vein tumor thrombus as independent predictors of early recurrence. Patients in the recurrence group demonstrated lower PLT levels, consistent with results from prior meta-analyses (13). In contrast, Han et al. (14) reported that higher preoperative PLT was associated with increased recurrence risk.

Elevated AFP levels (> 400 ng/mL) are a well-established adverse prognostic indicator in HCC and have been consistently confirmed as predictors of post-transplant recurrence (3, 15, 16). Ascites, as a manifestation of impaired hepatic function and portal hypertension, has been linked to poorer survival outcomes in patients with HCC and accelerated recurrence (11, 17). MVI is a critical risk factor for recurrence and metastasis after both curative resection and liver transplantation, and arterial peritumoral enhancement has been validated as a noninvasive imaging predictor of MVI in multiple studies (5, 18–20), consistent with these results. Similarly, portal vein tumor thrombus has been associated with increased risk of distant metastasis (21). Although clinical and conventional imaging indicators provide predictive value, their sensitivity and accuracy remain limited. To address this limitation, deep learning was introduced in the present study to extract high-dimensional imaging features from CT data, thereby enhancing predictive precision.

Most previous deep learning research used imaging features derived solely from arterial or venous phases (8, 22). However, each CT phase provides unique diagnostic information, and single-phase analysis may not capture the full spectrum of tumor characteristics. To overcome this limitation, this study adopted a multichannel deep learning approach, thereby improving the utilization of multiphasic CT features. The MP DL model achieved strong predictive performance, with an AUC of 0.934 in the test group, consistent with findings reported by Liao et al. (23). Furthermore, integration of the clinical-imaging model with the MP DL model resulted in the construction of the MD DL model, which achieved superior predictive performance, with an AUC of 0.985 in the test group, and demonstrated the highest sensitivity and specificity. These findings provide compelling evidence of the enhanced predictive value of multimodal approaches.

The use of MD DL models in recurrence prediction has been supported by several studies. Zhao et al. (24) developed MD DL and radiomics models using contrast-enhanced MRI and demonstrated their effectiveness in predicting early recurrence following curative hepatectomy. Similarly, a multicenter study reported that integrating radiomics, deep learning, and clinical data improved recurrence prediction following thermal ablation of HCC (25). Another multicenter investigation (26) indicated that a deep neural network model derived from multisequence MRI outperformed conventional criteria, such as the Milan criteria, for predicting early recurrence after liver transplantation.

We further compared our multimodal DL model with state-of-the-art methods for HCC prognosis prediction (27–29). This model was developed specifically for the stratification of early recurrence risk in liver transplant candidates using preoperative multiphasic CT and clinical data. Recurrence-free survival was used as the primary endpoint and resulted in competitive predictive performance with a simplified architecture. These results indicate that the proposed model has unique advantages and provides a clinically practical tool for preoperative risk assessment and early recurrence prediction.

Despite these advances, a major challenge remains the limited interpretability of deep learning, often referred to as the “black box,” which constrains clinical application. To address this limitation, this study used the SHAP method to quantify the contribution of each feature. This analysis showed that clinical variables and conventional imaging features made important contributions to the final prediction, while high−dimensional deep learning imaging features provided valuable complementary information. These results highlight the synergistic and complementary value of integrating clinical and imaging data in the MD DL model, which significantly improved predictive performance and robustness.

Several limitations should be acknowledged. First, this was a single-center retrospective study with a relatively small sample size. Accordingly, external multicenter validation is warranted to strengthen the clinical credibility and generalizability of the proposed model. Second, although data augmentation was applied to mitigate data scarcity and overfitting, the limited sample size may still have introduced bias. Expansion of the study group is essential for more comprehensive performance assessment. Third, although the ResNet architecture and attention mechanisms were incorporated, interpretability of the model was limited. Future research should focus on developing novel techniques to improve interpretability and to enhance understanding of the predictive mechanisms. Fourth, patients who received TACE, RFA, or PEI were excluded. Although this reduced imaging variability, it may limit the generalizability of our model to real-world transplant populations where bridging therapies are common. Future studies with more inclusive cohorts and diverse pretreatment histories are warranted to further validate the model’s clinical applicability.

In conclusion, the MD DL model integrating clinical data and multiphasic CT developed in this study, demonstrated high predictive accuracy, sensitivity, and specificity for early HCC recurrence after liver transplantation, supporting its potential clinical value in preoperative evaluation. The proposed model holds potential for preoperative risk stratification in liver transplant candidates, allowing individualized prediction of early HCC recurrence. This may facilitate clinical decision-making and improve perioperative risk evaluation, further underscoring its clinical value in transplant practice.

## Data Availability

The raw data supporting the conclusions of this article will be made available by the authors, without undue reservation.
